# Design of cell expansion processes for adherent‐growing cells with mDoE‐workflow

**DOI:** 10.1002/elsc.202200059

**Published:** 2023-04-25

**Authors:** Kim B. Kuchemüller, Ralf Pörtner, Johannes Möller

**Affiliations:** ^1^ Bioprocess and Biosystems Engineering Hamburg University of Technology Hamburg Germany

**Keywords:** early stage development, L929, mathematical process model, microcarrier culture, model‐assisted Design of Experiments (mDoE)

## Abstract

Adherent cells, mammalian or human, are ubiquitous for production of viral vaccines, in gene therapy and in immuno‐oncology. The development of a cell‐expansion process with adherent cells is challenging as scale‐up requires the expansion of the cell culture surface. Microcarrier (MC)‐based cultures are still predominate. However, the development of MC processes from scratch possesses particular challenges due to their complexity. A novel approach for the reduction of development times and costs of cell propagation processes is the combination of mathematical process models with statistical optimization methods, called model‐assisted Design of Experiments (mDoE). In this study, an mDoE workflow was evaluated successfully for the design of a MC‐based expansion process of adherent L929 cells at a very early stage of development with limited prior knowledge. At the start, the analytical methods and the screening of appropriate MCs were evaluated. Then, cause‐effect relationships (e.g., cell growth related to medium conditions) were worked out, and a mathematical process model was set‐up and adapted to experimental data for modeling purposes. The model was subsequently used in mDoE to identify optimized process conditions, which were proven experimentally. An eight‐fold increase in cell yield was achieved basically by reducing the initial MC concentration.

**Abbreviations**
Amm: ammonia
ATMP: advanced therapy medicinal products
BBD: Box–Behnken design
CCD: central composite design
CHO: Chinese hamster ovary
DAPI: 4′,6‐diamidine‐2‐phenylindole
DMEM: Dulbecco's Modified Eagle Medium
DoE: design of experiments
EDTA: ethylenediaminetetraacetic acid
FBS: fetal bovine serum
Glc: glucose
Gln: glutamine
Lac: lactate
LHSD: Latin hypercube sample design
LS: limiting substrate
MC: microcarrier
mDoE: model‐assisted Design of Experiments
PBS: phosphate buffered saline
PD: population doublings
SG: SYBR Green
VF: multiplication factor

## 1 INTRODUCTION

The application of adherent cells, mammalian or human, have gained increased attention in recent years. They are ubiquitous in the production of viral vaccines (human and veterinary), in gene therapy and in immuno‐oncology [1]. Especially advanced therapy medicinal products (ATMPs), which are divided into gene and cell therapeutics as well as tissue engineered products, relay to a large extent on adherent cells [2, 3, 4].

The development of a cell‐expansion process with adherent growing cell cultures possesses a particular challenge as scale‐up requires the expansion of the cell culture surface [1, 5]. In small scale, static culture dishes (e.g., well plates, T‐flasks, stacked plate systems) are used for cell expansion. For most of these systems monitoring and control of parameters such as temperature, pH, and oxygen supply is not established. As plates and flasks allow for a limited expansion only, scalable and instrumented bioreactor systems based on matrices to provide cell attachment have been introduced. These cover microcarriers (MCs) for use in standard stirred tank reactors, hollow fiber bioreactors, bioreactors for macrocarriers (e.g., fixed bed), and meander type bioreactors [6, 7].

MC‐based cultures are still predominate [8, 9, 10, 11]. However, the development of cell expansion processes from scratch has particular challenges due to the complexity of MC systems, their handling and characteristics and analytical methods. This may lead to immense efforts during process development, including challenging development procedures and long timelines [12].

A novel approach for the reduction of development times and costs of cell propagation processes is the combination of mathematical process models with statistical optimization methods, called model‐assisted Design of Experiments (mDoE). In contrast to classical experimental Design of Experiments (DoE), the process understanding is captured in a mathematical model which is used to evaluate experimental designs before they are performed in the laboratory. Recommended experiments are thus implemented in a significantly reduced number based on process understanding. To date, the successful application of mDoE has been demonstrated for the optimization of medium and feeding strategies for antibody‐producing Chinese hamster ovary (CHO) and yeast cells (*Saccharomyces cerevisiae*) [13, 14].

**PRACTICAL APPLICATION**

Adherent cells, mammalian or human, are ubiquitous for the production of viral vaccines, gene therapy and immuno‐oncology. For cell‐expansion processes microcarrier (MC)‐based cultures still predominate. However, the development of MC processes has a particular challenge due to their complexity. A novel approach for the reduction of development times and costs of cell propagation processes is the combination of mathematical process models with statistical optimization methods, called model‐assisted Design of Experiments (mDoE).

The study shows that mDoE concepts and the developed mDoE workflow are applicable for early stage process development with adherent‐growing cells. The mDoE workflow offers the advantage that knowledge can be developed iteratively, so that analytical methods can be established and only experiments with high knowledge gain are implemented. This ultimately leads to a faster realisation of the clinical development phases and thus to an earlier market entry of the biopharmaceutical.

In this study, the mDoE workflow explained below was evaluated for the design of a MC‐based expansion process of adherent‐grown L929 cells assuming a very early stage of development with limited prior knowledge. At the start, the analytical methods and appropriate MCs were selected and evaluated. Then, cause‐effect relationships (e.g., cell growth related to medium conditions) were  worked out, and a mathematical process model was set‐up and adapted to experimental data for modeling purpose. The model wasfinally used in mDoE to identify optimized process conditions, which have to be experimentally proven.

**FIGURE 1:**
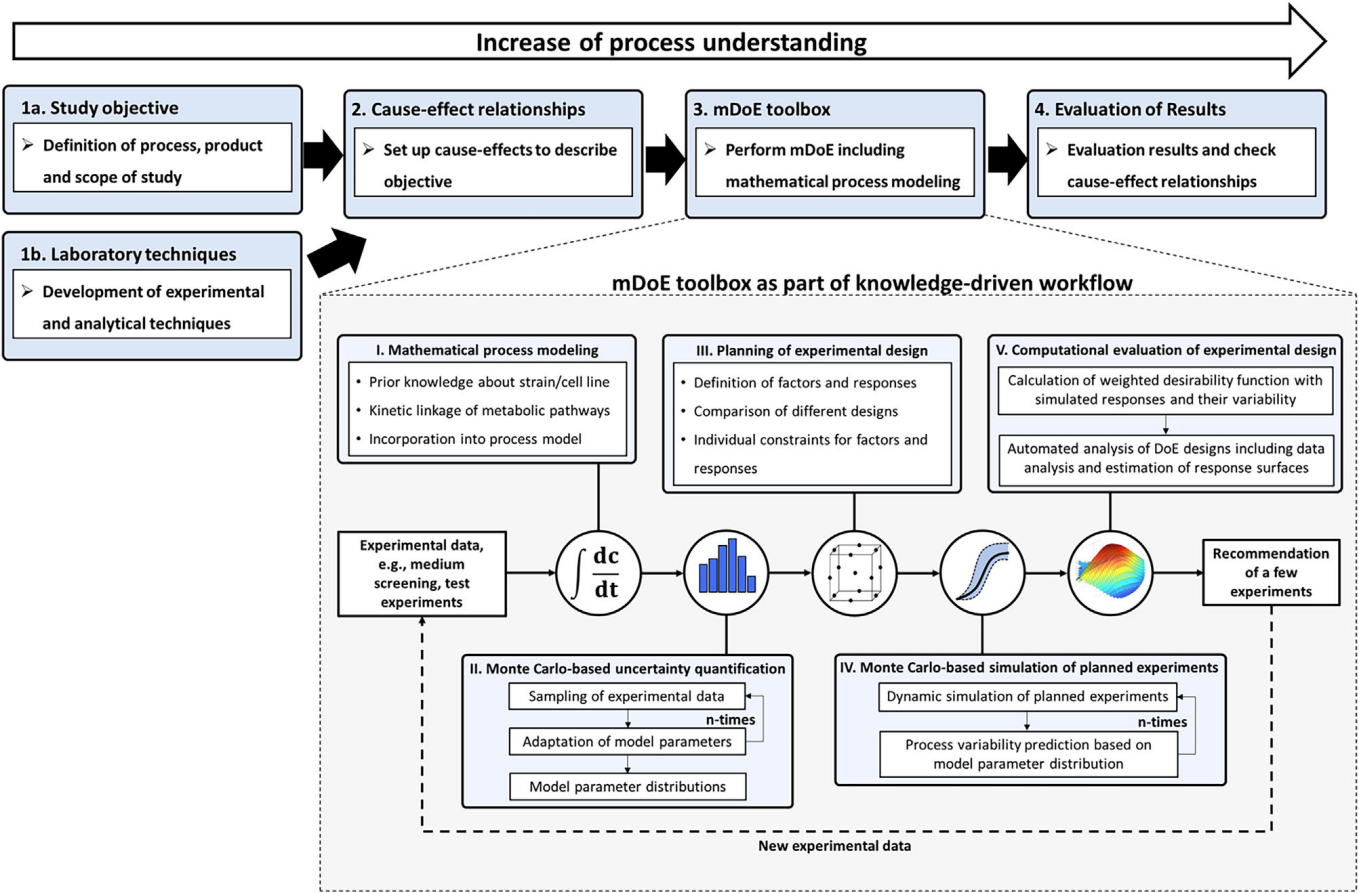
Integration of the model‐assisted Design of Experiments (mDoE) toolbox into a structural workflow. mDoE toolbox consisting of the combination of mathematical process models and Design of Experiments (DoE) under the consideration of model parametric uncertainty based on experimental variability, modified from [14].

In the following, the mDoE toolbox is introduced (see Figure 1). The general conditions and the objective are defined at the start of the mDoE‐workflow (“1a. Study objective”). More specifically, the product is defined including the desired expansion process and the final optimization goal (e.g., productivity, process runtime, statistical boundary conditions). Additionally, the general expansion process (e.g., bioreactor system, operation mode, cell line) and the main analytical methods need to be developed to enable an optimization study (“1b. Laboratory techniques”). Subsequently, the potential cause‐effects are set to define the influencing factors for the targeted optimization goal with respect to the desired objective (“2. Cause‐effect relationships”). These cause‐effect relationships can be based on prior knowledge obtained from literature, expert knowledge, and pre‐experiments. Based on our experience, a profound evaluation of the underlying dependencies and interactions is crucial in the beginning to avoid time‐consuming reiterations. During this step, data integrity and a plausibility check of the data quality with respect to the objective of the study should be performed (garbage in—garbage out phenomena). If necessary, the research objective needs to be adapted or further prior knowledge must be generated. Although prior knowledge must be generated at this point, the amount of data to be determined is much smaller compared to fully experimental workflows. Based on prior knowledge, assumptions are iteratively formulated to describe cause‐effect‐relationships, for example, to describe cell growth and cell metabolism.

In the following, the main parts of the mDoE software toolbox are briefly introduced (“3. mDoE toolbox”). Please see [14] for more details about the toolbox and its application.

In the first step of mDoE, the mathematical process is modeled based on the previously defined cause‐effect relationships (Figure 1, box I). Based on the mathematical model, the uncertainties of the model parameters are numerically calculated with Monte Carlo sampling under consideration of the experimental uncertainty (i.e., measurement error, Figure 1 box II). Therefore, the distribution of the model parameters reflects the process knowledge, meaning wide or multiple overlaying distributions identify not described effects and more narrow distributions mostly reflect well‐known effects. The third step is the planning of experimental settings using DoE algorithms (Figure 1, box 3). It is advantageous to use DoE for the planning of experimental settings since the factor combinations are efficiently planned and distributed in the space of potential experiments, that is, experimental space. Different experimental designs can be evaluated using the mathematical model and compared with each other without any experimental effort. Next, the bioprocess is simulated multiple times for each factor combination of the defined DoE using the determined parameter distributions. In other words, it is simulated how likely the dynamic behavior of cell growth and metabolism is in the bioprocess (Figure 1, box IV). These simulations are evaluated (see [14]) and the most appropriate settings are recommended to be experimentally performed. After the new experimental results are available, these data are evaluated (“4. Evaluation of results”) so that the process knowledge is expanded run by run and the defined cause‐effect relationships  are evaluated. Depending on the respective problem and/or the existing knowledge, individual steps of the workflow can be omitted.

## 2 MATERIALS AND METHODS

### 2.1 Cell lines, pre‐cultivation and passaging in T‐Flasks

In this study, adherent‐growing L929 cells (NCTC clone 929, ATCC, USA) were cultivated in static (e.g., T‐flasks) and in dynamic (e.g., shaken) bioreactors. For the static cultivations, cryo‐cultures containing 1 × 10^6^ cells were thawed and transferred to 10 mL phosphate‐buffered saline (PBS) (Carl Roth, Germany). The cell suspension was centrifuged at 4 min for 200 xg (Avanti J‐26SXP, Beckmann Coulter, USA) and the supernatant was removed. The cell pellet was resuspended in pre‐warmed Dulbecco's Modified Eagle Medium (DMEM) (PAN‐Biotech GmbH, Germany) and transferred to a cell culture T‐flask (Greiner Bio‐One, Austria) at a seed density of 5000 cells cm^−2^ in DMEM with 10 vol‐% fetal bovine serume (FBS) (FBS Superior, Biochron GmbH, Germany). The cells were cultivated at 37°C and 5 vol‐% CO_2_ in an incubator (HeraCell 150i, Thermo Fisher, USA). Depending on the experimental setup, the glucose (Sigma–Aldrich, USA) and glutamine (Lonza Group AG, Switzerland) concentrations were adjusted in the medium. Additional 1 vol‐% penicilin/streptomycin (Corning, USA) was added to the cultivation medium.

Reaching a confluence of 80%–90%, the cells were proteolytically solubilized from the growth surface with 1 vol‐% trypsin (Lonza Group AG, Switzerland) in PBS. For this, the medium was removed, and the T‐flask was washed twice with PBS (*V*_PBS_ = *V*_Medium_). Then, the trypsin solution was applied and incubated for 5–7 min. Enzymatic proteolysis was stopped by the addition of medium (*V*_Trypsin_ = *V*_Medium_), so that new cultivation systems were subsequently inoculated with the obtained cell solution.

### 2.2 Cultivation in shake flasks

Dynamic cultivations were performed in shake flasks (glass, Schott AG, Germany) with working volumes of 25, 40, and 60 mL cultivation medium (DMEM; see above) and different types and concentrations of MCs (*c*_MC_). Cells were inoculated at a cell density of 6000 cells cm^−2^. Compared to the static systems, the cell number was increased to compensate possible cell loss due to non‐attachment to the MCs, as previously described by [15]. The shake flasks were incubated for one night without agitation at the beginning and subsequently the shaker speed (GFL 3005, GFL, Germany) was set to 60 rpm (shaking diameter = 10 mm). For cell count determination and medium analysis, samples were taken every 24 h up to every 48 h. For bead‐to‐bead‐transfer, 100% fresh medium and 25%, 50%, 75%, or 100% fresh MC were added after 3, 5, or 7 days.

Experiments with different initial conditions were carried out (see Table 1). The initial concentration of glucose was varied in the range of 5–60 mmol L^−1^. This range was chosen based on [13], with the lower limit set 75% lower to best verify the small influence of glucose concentration. Glutamine concentrations typically vary over a wide range of 2–12 mmol L^−1^. The concentration range of MCs was varied, based on literature research, from 1 to 20 g L^−1^ [8, 18, 23]. It is important to underline that not all experiments were determined at the beginning, but were developed through the acquired knowledge.

**TABLE 1:** Microcarrier, glucose, glutamine, as well as cell concentration for the performed experiments in shake flasks for model‐assisted analysis of the cell behavior of L929 cells.

Number	*c*_MC_ (g L^−1^)	*c*_Glc_ (mmol L^−1^)	*c*_Gln_ (mmol L^−1^)	*X*_Inokulum_ (cells cm^−2^)
Experiments for modeling				
1	20	30	12	6000
2	20	60	12	
3	10	5.6	2	
4	10	25	12	
5	10	60	12	
6	5	60	12	
Verification of mDoE				
7	3	25	4	6000
8	1	25	4	

*Notes*: Experiment 1–6 were used for mathematical model development and experiment 7–8 were used for verification of the mDoE. Experiment 1 to 6 were built iteratively during process development by gaining further knowledge.

Abbreviation: mDoE, model‐assisted Design of Experiments

Based on experiments 1 to 6, the behavior of the L929 cells was analyzed and the mathematical model was built. The remaining two experiments (number 7 and 8) were used to verify the mDoE.

### 2.3 Microcarriers and vessel siliconization

As MCs, Cytodex 3 (GE HealthCare, USA) and SoloHill (Sartorius, Germany) MCs were used. SoloHill MCs included Hillex II, Plastic, PlasticPlus, StarPlus, FACT III, and CollagenCoated. MCs were prepared as described in the manufacturers protocols.

Siliconization of all glass culture vessels was necessary to avoid adhesion of the MCs to the vessel wall. For this purpose, a few milliliter of Sigmacote (Sigma–Aldrich, USA) were transferred to the respective vessel under a fume hood and rotated and swirled at regular intervals to distribute the solution. The respective vessel was then rinsed with ultrapure water and dried overnight in a 60°C heating oven (Thermo Scientific Heratherm, ThermoFisher Scientific, USA).

### 2.4 Analytics

#### 2.4.1 Cell harvest and lysis

Various enzymatic as well as cell lysing methods came into question for the separation of cells from MCs. For all methods, 1 mL sample was taken, and the MCs were sedimented (1–3 min, depending on the respective MC). The supernatant was collected and stored for suspension cell count determination. Subsequently, the respective MC cell pellet was washed 2 times with PBS or with PBS + 2 mmol L^−1^ ethylenediaminetetraacetic acid (EDTA). For cell harvesting, two different enzymatic methods were used. For both methods, the samples were incubated with enzyme solution for 5–7 min. The first enzyme solution is based on a 1 vol‐% trypsin mixture in PBS, while the second is composed of a 1:1 mixture of 0.25 vol‐% trypsin in PBS and 0.02 vol‐% EDTA in PBS. Further instructions can be found in [15].

For the IGEPAL method, the protocol from [16] was adopted, but no sieve was used.

#### 2.4.2 Cell counting

##### Counting of cell nuclei

To determine the cell count, three different methods were eligible.

- Cell nuclei were stained by propidium iodide (Sigma–Aldrich, USA) and quantified using a flow cytometer (CytoFLEX, Beckman Coulter, USA) with the 585/42 filter and a 488 nm laser. Debris were excluded using SSC‐A versus FSC‐A gating and doublets were excluded with FSC‐H versus FSC‐A gating.
- A Z2 Coulter Particle Count and Size Analyzer (Beckmann Coulter, USA) was used for cell counting. For measuring, a total volume of 10 mL was diluted with PBS and 2 mmol L^−1^ EDTA according to the expected cell concentration.
- For quantification of fluorescence by the SYBR Green I (SG) (Sigma–Aldrich, USA) method, 1 mL of sample was centrifuged for 4 min at 200 xg, and the supernatant was removed and replaced with PBS. Subsequently, fluorescence measurements were performed in a black 96‐well plate (Corning, Germany) threefold using a microplate reader (Tecan Infinite Nano +, Tecan, Switzerland). Further details can be found in [17].

#### 2.4.3 Determination of the distribution of cells on microcarriers

Fluorescence staining with 4′,6‐diamidine‐2‐phenylindole (DAPI) (Carl Roth, Germany) was performed to assess cell growth on MCs. Samples were centrifuged at 200 x g for 3 min, washed twice with PBS and resuspended in 70 vol‐% ethanol. Intermediate storage was performed in a −20°C freezer (Bauknecht, Germany).

For microscopy, the ethanol supernatant of the samples was removed and replaced with PBS + 1 vol‐% Triton X‐100 + 0.1 vol‐% DAPI. The fluorescent dye was incubated at room temperature in the dark for a period of 5 min. Subsequently, 50 μL of sample was pipetted onto a slide with coverslip. Microscopy was performed at 358 nm using a violet filter on the Eclipse 80i fluorescence microscope.

#### 2.4.4 Quantification of glucose, glutamine, lactate and ammonia

Concentrations of glucose (*c*_Glc_), glutamine (*c*_Gln_), and lactate (*c*_Lac_) were measured with the YSI 2900D (Yellow Springs Instruments) biochemistry analyzer. The concentration of ammonia (*c*_Amm_) was determined with an enzymatic test kit (AK00091; NZYTech, Portugal).

### 2.5 Mathematical methods

#### 2.5.1 Estimation of model parameters

Least square methods were used to determine the specific model parameters, which were used as initial values to determine the expected process variability. For this purpose, the model parameters were varied 1000‐fold based on the experimental uncertainty (e.g., measurement error) and thus the measurement errors were simulated. Please see [13, 14].

#### 2.5.2 Computational evaluation of experimental designs

Common experimental designs as central composite (CCD), Box–Behnken (BBD), D‐optimal, I‐optimal and Latin hypercube sample design were created using the mDoE toolbox. The experimental factor combinations were simulated n‐times considering the parameter distribution function (Monte Carlo‐based), and the averaged target variables as well as their variability (difference of the 90% and 10% quantiles) were calculated. Subsequently, the averaged target variables and the variability were summarized in a desirability function. Please see [14] for further details.

## 3 RESULTS AND DISCUSSION

The aim of this study is the evaluation of the mDoE workflow at a very early stage of development without extensive prior knowledge, as outlined in Figure 1. As case study, the design of an MC‐based expansion process of adherent‐grown L929 cells is investigated. At first, the analytical methods as well as the cultivation conditions were defined and cause‐effect relationships are investigated as basis for the adaptation of a mathematical process model. Then, the mDoE methods were used to evaluate different experimental designs and to optimize cultivation conditions with respect to the maximum cell yield.

### 3.1 Elaboration and evaluation of relevant analytical and cultivation methods

Analytical methods, especially for cell detachment and cell count, and the screening of appropriate MCs are the basis for the development of an expansion process for adherent growing cells and the application of model‐assisted process development methods [10, 18, 19]. Therefore, the challenges in the development of a cell count method and the MC screening are shown in the following subsections.

#### 3.1.1 Quantification of cell number

Precise determination of the number of cells attached to a MC is still difficult and a number of different techniques have been suggested [10, 19]. The suitability of a specific method is case‐specific. Therefore, a screening and evaluation of different methods was performed.

**FIGURE 2:**
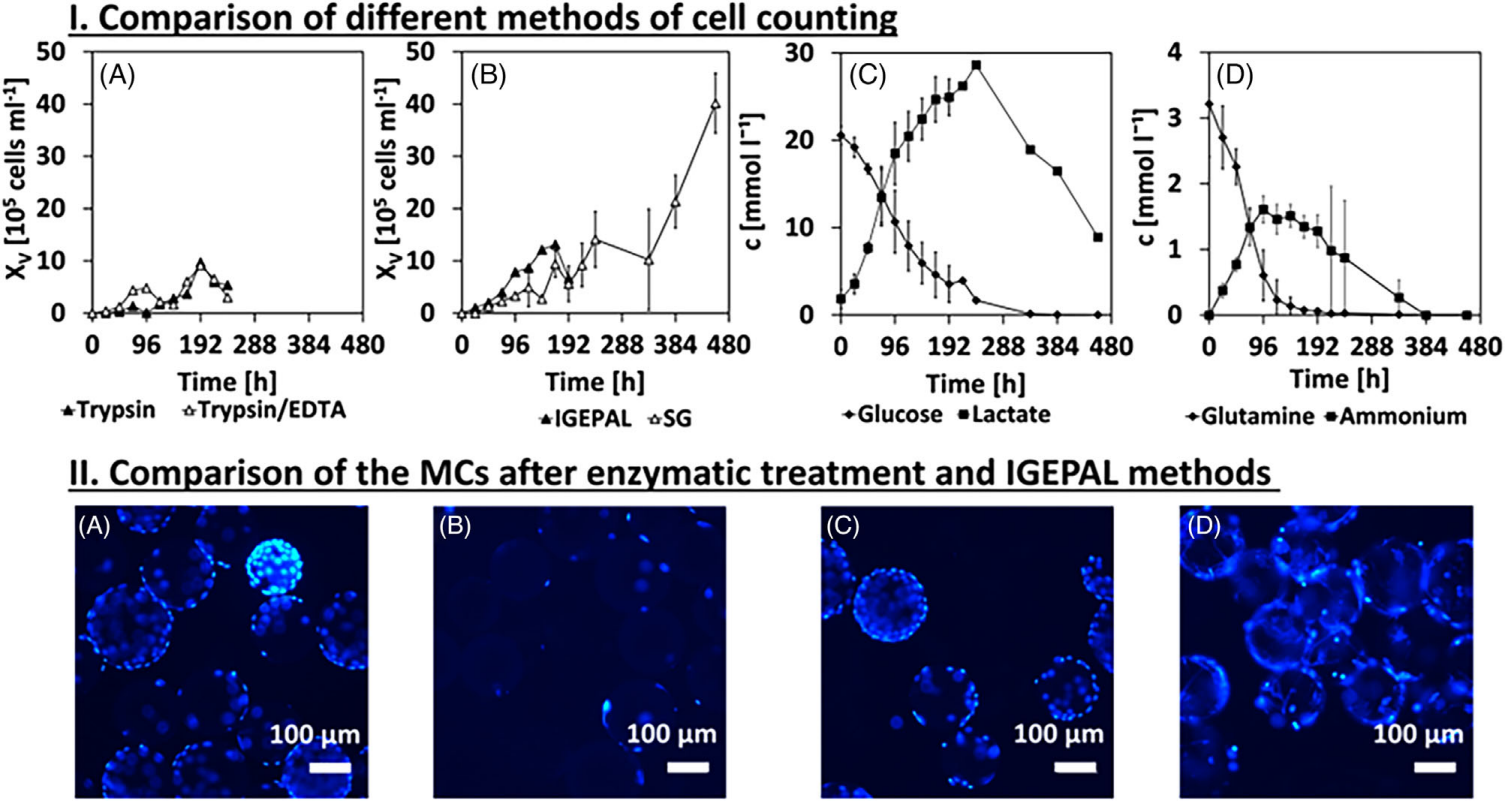
(I) Comparison of different methods for cell counting. Shown are determined cell numbers for two enzymatic methods (A), a cell lysis method (B, IGEPAL), and a photometric method that works without detaching cells from MCs (B, SYBR Green [SG]). In addition, the averaged substrate and metabolite courses (C, D) to the cell courses are illustrated. (II) An untreated microcarrier sample (A) was compared to IGEPAL method (B) and after application of the enzymatic method (C, trypsin; D, trypsin/EDTA) by fluorescence microscopy. Samples were collected after 72 h of cultivation.

As can be seen in Figure 2I, four different methods for cell count measurement are compared for the same cell cultivation process (see Sections 2.4.1 and 2.4.2). Measurements with the particle counter, used by enzymatic methods, can only be carried out in a certain concentration range, which is especially difficult to achieve at the beginning of cultivations (Figure 2IA). Using the SG method, rather constant cell growth is measured until day 20 (Figure 2IB). Hence, glutamine is fully consumed after 8 days and glucose after 14 days (Figure 2IC,D). Even if consumption of lactate is observed in some cell lines (e.g., CHO cells) after glucose consumption, such a lactate shift has so far only been documented in stationary growth phase [20] and is not sufficient for renewed cell growth. The results of the IGEPAL method (Figure 2IB) seem to be meaningful and as expected.

Fluorescence microscopy, as shown in Figure 2IIA,C,D, confirmed that no complete detachment of cells occurred for enzymatic and SG method. Using the IGEPAL method, only a few isolated cells remained on the MCs (Figure 2IIB). Therefore, in the remainder of this paper, the IGEPAL method will be used to count the cells. The trypsin/EDTA method is used to harvest the cells, as a comparatively high yield can be obtained. Nevertheless, it must be noted that a complete detachment cannot be achieved with enzymatic methods.

#### 3.1.2 Microcarrier screening

**FIGURE 3:**
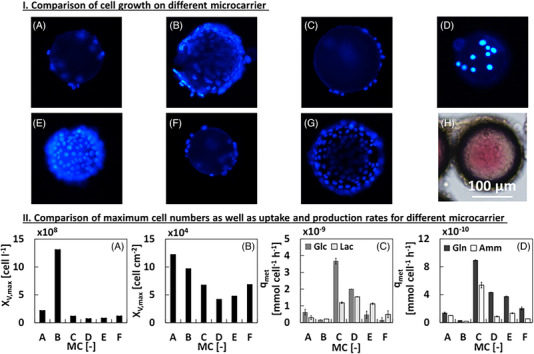
(I) Fluorescence microscopic image of the different microcarriers (MCs) (*c*_MC_ = 5 g L^−1^): (A, Collagen Coated; B, Cytodex 3; C, FACT; D, Plastic; E, PlasticPlus; F, StarPlus; G, Hillex; and H, Hillex with transmitted light) after 72 h of cultivation in a microtiter plate. (II) Maximum cell numbers in relation to volume (A) and growth area (B) as well as uptake and production rates (C,D) during the exponential phase of each MC (A, Collagen Coated; B, Cytodex 3; C, FACT; D, Plastic; E, PlasticPlus; F, StarPlus). The total cultivation period was 264 h, whereby maximum cell growth could be reached earlier in cultivation period. A MC concentration of 5 g L^−1^ was used in each cultivation.

L929 cells were cultivated on a range of commercially available MCs in a microtiter plate for 72 h. Subsequently, the MCs were qualitatively analyzed by DAPI staining (Figure 3I), whereby cells did not adhere and proliferate on the microtiter plate (not shown). Cytodex 3 (Figure 3IB), PlasticPlus (Figure 3E), and Hillex MCs (Figure 3IG) were almost completely overgrown, whereas hardly any cells had attached on the remaining MCs. However, the Hillex MCs absorbed the phenol red indicator present in DMEM growth medium (Figure 3IH).

All MCs (w/o Hillex), were cultured in shake flasks to determine the growth performance in long‐term experiments. The highest cell number related to the culture volume was obtained after 144 h for the Cytodex 3 MC with 1.3 × 10^6^ cells mL^−1^ (Figure 3IIAB), where with the other MCs significantly lower cell numbers per volume were achieved.

Nevertheless, a similar specific maximum growth rate was achieved in the exponential growth phase (*μ* = 0.03 h^−1^) (data not shown). Only the Plastic MC exhibited a lower specific maximum growth rate of *μ* = 0.016 h^−1^ (data not shown). Due to the different surface areas of the MCs, the yield was additionally considered in relation to the growth surface (Figure 3IIB). The Collagen Coated MCs (Figure 3IIB,B) reached a 0.2 × 10^4^ cells cm^−2^ higher cell number than Cytodex 3. However, the Collagen Coated MCs reaches this maximum cell number only after 192 h instead of 144 h. The values of uptake and production rates (Figure 3IIC,D) are in a comparable range to literature [15]. In total, it is surprising what a strong influence the choice of a MC has on cell growth and metabolism. Lower attachment results in decreased uptake and production rates. Overall, the Cytodex 3 MCs were further used in this study.

### 3.2 Working‐out of cause‐effect relationships

As basis of the mathematical process model, potential cause‐effect relationships need to be worked out (see Figure 1). No literature data was available for L929 cell growth on MCs at the beginning. Therefore, experiments were performed iteratively, using the generated knowledge of the performed experiment as a starting point for the development of the subsequent experiments. Typical medium components as *c*_Glc_ and *c*_Gln_ with a constant *c*_MC_ were examined, whereby no dependency of these factors on the maximal cell number was observed (Figure S1). Both substrates were always present in excess under the given experimental settings and no limitation was identified. No influence of the different settings of the substrate concentrations was detected either. In both cultivations, a cell number of 2.7 ± 0.07 × 10^9^ cells L^−1^ was achieved after a maximum of 192 h, with none of the substrates depleted at this time. Although high concentrations of lactate of 20 mmol L^−1^ and ammonia of 8 mmol L^−1^ were tested, cell growth was not inhibited. Growth inhibition is characterized by steadily reduced growth, so that a transition to stationary phase would be observed first rather than a direct transition to the death phase. Therefore, metabolite inhibition was ruled out as reason for the stopped cell growth after 192 h of cultivation. Common amino acids were measured to identify possible limiting factors (Figures S2–S3), and no limitation of amino acids was determined at all. Therefore, it was presumed that an unknown limiting substrate is influencing cell growth (e.g., serum components).

With consideration of two further experiments (shown in Figure S4), *c*_Glc_, *c*_Gln_, and *c*_MC_ were varied. Again, no effect of *c*_Glc_ and *c*_Gln_ on the growth rate was detected. Based on these experiments, it can be concluded that as long as both substrates are present, no growth declining effect based on substrate consumption seems to occur. Additionally, the MC concentration does affect growth. This becomes clear when looking at population doublings (PD), multiplication factor (VF), maximum cell number and the time of reaching the maximum cell number *t_X,V_*_,max_ as a function of MC concentration (see Table 2).

**TABLE 2:** Comparison of specific characteristic values, like maximum cell number (*X_V_*_,max_), time of reaching the maximum cell number (*t_X,V_*_,max_), population doublings (PD), and multiplication factor (VF) as a function of microcarrier (MC) concentration.

Number	*c*_MC_ (g L^−1^)	*X_V_*_,max_ (cells cm^−2^)	*t_X_*_,_ *_V_*_,max_ (h)	PD (–)	VF (–)
2	20	5 × 10^4^	192	3 ± 0.13	8.3 ± 0.5
4	10	5.2 × 10^4^	144	3 ± 0.15	8.3 ± 0.9
6	5	6.7 × 10^4^	144	3.8	14
7	3	22.5 × 10^4^ ± 10	216	5.2	37.5
8	1	39.8 × 10^4^ ± 39	216	6.1	66.3

*Note*: Numbers correspond to performed experiments, which specific initial conditions are explained in Table 1.

The highest cell‐area specific cell yield was obtained in the experiment with the lowest MC concentration (*c*_MC_ = 1 g L^−1^), with 6.7 × 10^4^ cells cm^−2^. In contrast, at the highest MC concentration (*c*_MC_ = 20 g L^−1^), the lowest cell concentration of 5 × 10^4^ cells cm^−2^ was achieved. In addition, the time of reaching the maximum cell number increased from 144 to 192 h. It is possible that at high MC concentration, cells were prevented from adhering by MCs bumping against each other, so that they required a longer period to expand.

### 3.3 Building the mathematical model

The previously formulated cause‐effect relationships are used to adapt an existing model for suspension‐growing mammalian cell lines [13] to describe the growth and metabolism of adherent‐growing cells in dynamic cultivations. The model initially formulated for suspension cells was expanded by additional equations to describe the initial attachment phase of cells onto the MCs and the incorporation of growth limitation due to contact inhibition and cell metabolism as shown in Table S1 “Experiments for modeling”. The modification was performed iteratively with model‐assisted data analysis. The main substrates glucose, glutamine, and an unknown limiting substrate (*c*_LS_) are linked with the major metabolites lactate (*c*_Lac_) and ammonia (*c*_Amm_) to describe the growth behavior of the cells. The introduction of an unknown limiting substrate summarizes all components that lead to limitation, but which cannot be directly measured (e.g., serum components). $c_{\mathrm{LS}}\;$and the metabolites are used to describe the cells growing on the MCs. The cell growth ($X_{V}$) and the growth of the cells in suspension (*X*_Sus_) are expressed with the kinetic parameters $K_{S,\mathrm{LS}}$ and $K_{d,\mathrm{LS}}$, a maximum growth rate μ_max_, an attachment constant *k*_att_ and a minimum $\mu_{d,\min\;}$and maximum death rate $\mu_{d,\max}$. The implementation of$\;k_{\mathrm{att}}$ is essential for the description of the initial cell attachment phase. For the first 24 h of cultivation, *k*_att_ is maximal. Since cells that detach in the initial phase are apoptotic and thus lose their adherent functions, $k_{\mathrm{att}}\;$= 0 after 24 h [21]. Cells that are still in the liquid phase after the initial attachment phase of 24 h are defined as *X*_Sus_.

Since no growth limitation related to *c*_Glc_ and *c*_Gln_ was detected for the experimental conditions applied in this study, these substrates were not considered as limiting components in the growth kinetics. Therefore, the calculation of the specific growth rate *μ* and the specific death rate *μ_d_* is based on the concentration of the limiting component *c*_LS_ solely. This concept has been applied successfully in [22]. It should be noticed that this effect is based on the used media and applied conditions and was not intended in the study design.

The cell‐specific uptake rates of glucose and glutamine depend on the ratio of growth to cell yield, $Y_{X/\mathrm{Glc}}$ for glucose and $Y_{X/\mathrm{Gln}}$ for glutamine. The concentrations of lactate and ammonia are proportional to the uptake rates of glucose (*q*_Glc_) and glutamine (*q*_Gln_) and they are linked to the yield coefficients. To account for a limiting space on the MCs, the remaining space was expressed as the proportion of the maximal cell number on the MCs$\;X_{V,\max}$ as $\frac{X_{V,\max}-\;X_{V}}{X_{V,\;\max}}$. $X_{V,\;\max}$ was calculated according to [23]. At the beginning of cultivation, $\frac{X_{V,\max}-\;X_{V}}{X_{V,\;\max}}$ is high meaning that much space is left on the MCs. Due to cell growth, less space becomes available on the MCs and the cell growth rate decreases, which is reflected with a decreasing $\frac{X_{V,\max}-\;X_{V}}{X_{V,\;\max}}$ . The mathematical model equations are shown in the following equations:

(1) $$\;\frac{dX_{t}}{dt}=\;\mu \cdot X_{V}\;-\;K_{\mathrm{Lys}}\cdot (X_{t}\;-\;X_{V})\;$$

(2) $$\;\frac{dX_{V}}{dt}=\;\left(\mu \;-\;\mu_{d}\;\right)\cdot X_{V}\;+k_{\mathrm{att}}\cdot \frac{X_{V,\max}-\;X_{V}}{X_{V,\;\max}}\cdot X_{\mathrm{Sus}}$$

(3) $$\mathrm{with}\;\mu \;\;=\;\mu_{\max}\cdot \frac{c_{\mathrm{LS}}}{c_{\mathrm{LS}}\;+\;K_{S,\mathrm{LS}}}\cdot \frac{X_{V,\max}-\;X_{V}}{X_{V,\;\max}}$$

(4) $$\mathrm{and}\;\mu d\;\;=\;\mu_{d,\min\;}+\;\mu_{d,\max}\cdot \frac{c_{\mathrm{LS}}}{c_{\mathrm{LS}}\;+\;K_{d,\mathrm{LS}}}$$

(5) $$\frac{dX_{\mathrm{Sus}}}{dt}\;=\;\mu_{d}\;\cdot X_{V}-k_{\mathrm{att}}\cdot \frac{X_{V,\max}-\;X_{V}}{X_{V,\;\max}}\cdot X_{\mathrm{Sus}}$$

During cell attachment: *t* < 20 h: *k*_att_ = *k*_att, max_; *t* ≥ 20 h: *k*_att_ = 0

(6) $$\;\frac{dc_{\mathrm{Glc}}}{dt}=-\;q_{\mathrm{Glc}}\;\cdot X_{V}$$

(7) $$\mathrm{with}:\;\;q_{\mathrm{Glc}}=\;\frac{\mu }{Y_{X/\mathrm{Glc}}}\cdot \frac{c_{\mathrm{Glc}}}{c_{\mathrm{Glc}}\;+\;k_{\mathrm{Glc}}}\cdot \frac{X_{V,\max}-\;X_{V}}{X_{V,\;\max}}$$

(8) $$\;\frac{dc_{\mathrm{Gln}}}{dt}=\;-q_{\mathrm{Gln}}\cdot X_{V}$$

(9) $$\mathrm{with}\;\;q_{\mathrm{Gln}}=\frac{\mu }{Y_{X/\mathrm{Gln}}}\;\cdot \frac{c_{\mathrm{Gln}}}{c_{\mathrm{Gln}}\;+\;k_{\mathrm{Gln}}}\cdot \frac{X_{V,\max}-\;X_{V}}{X_{V,\;\max}}$$

(10) $$\;\frac{c_{\mathrm{LS}}}{dt}=-\;q_{\mathrm{LS}}\;\cdot X_{V}$$

(11) $$\mathrm{with}\;\;q_{\mathrm{LS}}=q_{\mathrm{LS},\max}\;\cdot \frac{c_{\mathrm{LS}}}{c_{\mathrm{LS}}+\;k_{\mathrm{LS}}}\;$$

(12) $$\;\frac{dc_{\mathrm{Lac}}}{dt}=Y_{\mathrm{Lac},\mathrm{Glc}}\;-\;q_{\mathrm{Glc}}\cdot X_{V}\;$$

(13) $$\;\frac{dc_{\mathrm{Amm}}}{dt}=\;Y_{\mathrm{Amm},\mathrm{Gln}}\cdot q_{\mathrm{Gln}}\cdot X_{V}$$

The parameters were adapted based on the previously presented experiments, shown in Table S2. The adaptation of this mathematical process model is shown in Figure S5. The course of *X_V_* is represented for the exponential growth phase as well as the death phase with an accuracy of *R* ^2^ = 0.80. The changes of *c*_Glc_ and *c*_Gln_ are represented with an *R* ^2^ > 0.9, whereas the simulations of *c*_Lac_ and *c*_Amm_ are characterized by an *R* ^2^ > 0.4. A metabolic shift from lactate production to lactate uptake was observed in the experimental data set (Figures S1B,E as well as S4B,E). However, the shift is neither coupled to the transition of the exponential phase to the stationary phase nor to specific substrate concentrations. Thus, no assumptions were formulated for implementation in the mathematical process model. Accordingly, the time course of the lactate concentration is inaccurately represented. A similar behavior can be seen for the course of *c*_Amm_. However, since the metabolites do not cause inhibition and the trajectories are mapped, the low coefficient of determination is considered sufficient and the mathematical model was used for the mDoE. Additionally, based on the six experiments from Table 1, model uncertainties were considered, as initial values were varied 1000‐fold by 15% (Monte Carlo sampling). Uncertainty quantification guaranteed that the mathematical model reflects the experimental behavior for scattered initial values.

### 3.4 mDoE toolbox

#### 3.4.1 Comparison of different DoE plans

**FIGURE 4:**
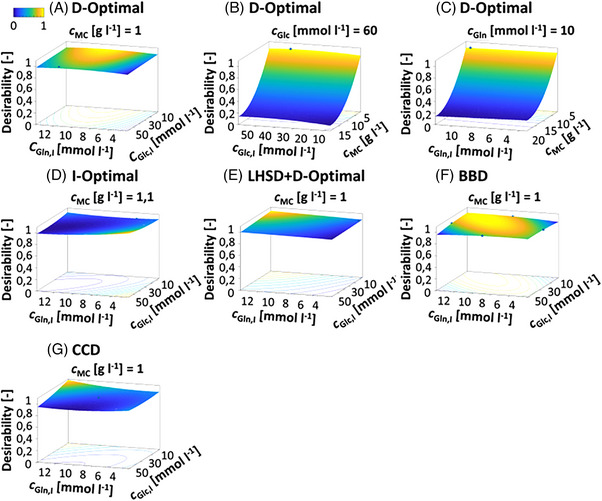
RSM graphs of desirability function (calculated by maximum cell number) for optimization *c*_Glc,I_ and *c*_Gln,I_ of a cultivation process in batch mode. A, D‐optimal; I‐optimal, LHSD + D‐optimal experimental design, BBD, and CCD were compared. The blue balls are factor combinations. For three factors, one of the factors was fixed at its optimum in the RSM graphs, the set value is noted above the RSM graph. BBD, Box–Behnken design; CCD, central composite design; LHSD, Latin hypercube sample design.

Using the mDoE toolbox in the proposed knowledge‐driven workflow (Figure 1), a justification regarding a suitable experimental design for bioprocess optimization purpose can be made (Figure 4). Therefore, DoEs can be planned, and the outcome of all factor combinations are simulated. Furthermore, different DoE designs can be compared. Here, a D‐optimal, I‐optimal, LHSD + D‐optimal experimental design as well as CCD and BBD were used to design the factor combinations which were further simulated. The initial concentration of glucose (*c*_Glc,I_) was varied in the range of 5–60 mmol L^−1^. This range was chosen based on literature [13], with the lower limit set downward by 75% to best check the small influence of glucose concentration. The initial Glutamine concentration (*c*_Gln,I_) typically vary over a wide range of 2–12 mmol L^−1^, so this was no longer changed. *c*_MC_ was varied in the range of 1–20 g L^−1^. The cell number was chosen as aim of optimization. The desirability function was calculated by maximizing *X_V_* shown for all experimental designs in Figure 4A‐G.

For the five experimental designs, maximum area‐specific cell yield was achieved at an MC concentration of 1 g L^−1^ regardless of the initial glucose and glutamine concentrations. The maximal desirability varied for all experimental designs by approximately 5%. For the CCD and BBD, 20 and 24 instead of 16 factor combinations were required to generate the experimental designs compared with the optimal and LHSD + D‐optimal experimental designs. Thus, in an experimental implementation, the optimal and LHSD + D‐optimal experimental designs would be recommended due to a reduced experimental workload. With the D‐optimal experimental design, there is additionally little variation in generation quality (not shown), making a D‐optimal experimental design preferable. Although the small influence of *c*_Glc_ and *c*_Gln_ in the mathematical model was predictable, these two factors were examined to show differences when comparing different experimental designs.

#### 3.4.2 Output of the mDoE methods

**FIGURE 5:**
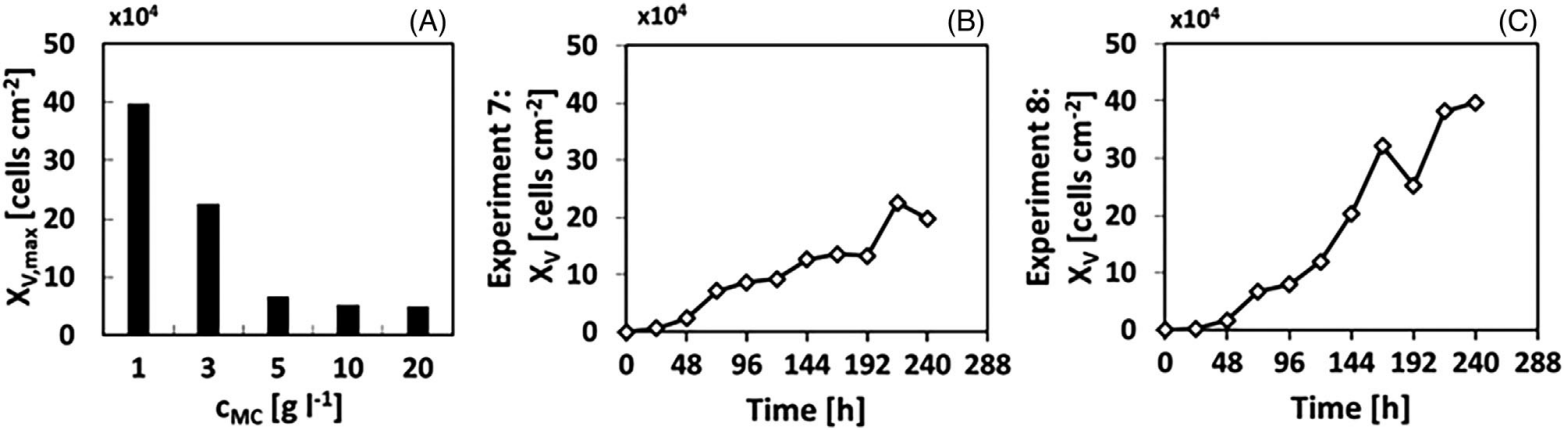
(A) Maximal cell concentration in dependency of the microcarrier concentration (based on experiments in Table 1). (B) Growth curve of L929 cells for experiments 7 (see Table 1, *c*_MC_ = 3 g L^−1^) and, (C) Growth curve of L929 cells for experiments 8 (see Table 1, *c*_MC_ = 1 g L^−1^). For both experiments, *c*_Glc,I_ = 25 mmol L^−1^ and *c*_Gln,I_ = 4 mmol L^−1^.

Based on the mDoE‐toolbox, optimized experimental conditions with a minimum *c*_MC_ = 3 g L^−1^ and 1 g L^−1^ and a mean *c*_Glc,I_ = 25 mmol L^−1^ and *c*_Gln,I_ = 4 mmol L^−1^ were recommended (see Table 1, experiments 7 and 8). As can be seen in Figure 5A, reducing *c*_MC_ to 1 g L^−1^ resulted in a six‐fold increase in cell yield compared to cell yields at a *c*_MC_ of 5 g L^−1^ (Figure 5A). Compared to the cell yield at a *c*_MC_ of 20 g L^−1^, even an eight‐fold increase was achieved. The individual growth curves of both cultivations are shown in Figure 5B  (*c*_MC_ = 3 g L^−1^) and Figure 5C (*c*_MC_ = 1 g L^−1^). For both cultivations, the general growth behavior was as expected, and the maximal cell number was reached after 240 h.

## 4 CONCLUSION

The aim of this study was to verify to what extent the mDoE workflow were applicable for early stage process development with adherent‐growing cells. Especially at the beginning of a process development, there is a demand for certainty of prediction. The mDoE workflow offers the advantage that knowledge can be developed iteratively, so that only experiments with high knowledge gain are implemented. At the same time, the number of experimental runs can be reduced. This ultimately leads to a faster implementation of the clinical development phases and thus to an earlier market entry of the biopharmaceutical. For the development of a cell expansion process with adherent‐growing L929 cell, the mDoE workflow enabled in‐depth analysis of cell behavior. The required analytical and cultivation methods were established in a very short time and knowledge about cell behavior was generated. Through the results of 6 shake flask experiments, an eight‐fold increase in cell yield was achieved in this study. Further research is needed to tightly integrate cultivation experiments and corresponding virtual mathematical models with regulatory guidelines. Therefore, scientists need to be trained interdisciplinary in classical bioprocess engineering, real‐world industrial applications in cell cultivation, digitization solutions, and advanced analytics.

## CONFLICT OF INTEREST STATEMENT

The authors declare no conflicts of interest.

## Supporting information

Supporting Material

## Data Availability

The data that support the findings of this study are available from the corresponding author upon reasonable request.
