# Supplementary material for: Design of cell expansion processes for adherent‐growing cells with mDoE‐workflow
Source: Eng Life Sci. 2023 Apr 25;23(5):e2200059. doi: 10.1002/elsc.202200059 (PMC10158623; doi:10.1002/elsc.202200059)
Supplement: Supplementary file 1 — Supporting Material [file ELSC-23-e2200059-s001.pdf]

**Supplements to: Design of cell expansion processes for adherent-growing cells with mDoE-workflow**

Kim B. Kuchemüller[1], Ralf Pörtner[1], Johannes Möller[1]

[1]Hamburg University of Technology, Bioprocess and Biosystems Engineering, Hamburg, Germany

**Correspondence:** Dr. Johannes Möller (johannes.moeller@tuhh.de). [1]Hamburg University of Technology, Bioprocess and Biosystems Engineering, Denickestraße 15, 21073 Hamburg, Germany

**Keywords:** Microcarrier culture, early-stage development, mathematical process model, model-assisted Design of Experiments (mDoE), L929

**Abbreviations: Amm**, Ammonia; **ATMP**, Advanced Therapy Medicinal Products; **BBD**, Box-Behnken Design; **CCD**, Central Composite Design; **CHO**, Chinese Hamster Ovary; **DAPI** 4',6-diamidine-2-phenylindole; **DMEM**, Dulbecco's Modified Eagle Medium; **DoE,** Design of Experiments; **EDTA,** Ethylenediaminetetraacetic acid; **FBS**, Fetal Bovine Serum; **Glc**, Glucose; **Gln**, Glutamine; **Lac**, Lactate; **LHSD**, Latin Hypercube sample design; **LS**, Limiting substrate; **MC**, Microcarrier; **mDoE** model-assisted Design of Experiments; **PBS,** Phosphate buffered saline; **PD**, Population doublings; **SG**, SYBR Green; **VF**, Multiplication factor

**Supplementary Table 1** Implemented boundary condition of the mathematical process model to consider the influence of MC concentration.

| $c_{MC}$ [g l$^{-1}$] | Boundary condition |
|---|---|
| Experiments for modelling | |
| 20 | $\mu = 0.6x\mu_{max}$ |
| | $q_{ls,max} = 0.3xq_{ls,max}$ |
| | $Y_{X,Glc} = 1.4xY_{X,Glc}$ |
| 5 | $Y_{X,Glc} = 0.5xY_{X,Glc}$ |
| | $Y_{X,Gln} = 0.5xY_{X,Gln}$ |
| Verification of mDoE | |
| 3 | $q_{LS,max}$=0.5 $q_{LS,max}$ |
| | $Y_{X,Glc}$=0.5 $Y_{X,Glc}$ |
| | $Y_{X,Gln}$=0.5 $Y_{X,Gln}$ |
| 1 | $\mu_{max}$=1.2 $\mu_{max}$ |
| | $Y_{X,Glc}$=0.5 $Y_{X,Glc}$ |
| | $Y_{X,Gln}$=0.5 $Y_{X,Gln}$ |

**Supplementary Table 2** Parameters of the mathematical process model as well as the associated start values and resulting values.

| Parameter | Unit | Starting value | Adapted value |
|---|---|---|---|
| $\mu_{d,max}$ | $h^{-1}$ | 0.02 | 0.0064 |
| $\mu_{d,min}$ | $h^{-1}$ | 0.003 | 0.0002 |
| $\mu_{max}$ | $h^{-1}$ | 0.02 | 0.026 |
| $K_{att,max}$ | $h^{-1}$ | 0.03 | 0.4 |
| $K_{d,LS}$ | mmol $l^{-1}$ | 0.005 | 0.065 |
| $K_{Glc}$ | mmol $l^{-1}$ | 0.19 | 0.03 |
| $K_{Gln}$ | mmol $l^{-1}$ | 2.5 | 0.61 |
| $k_{s,LS}$ | mmol $l^{-1}$ | 0.01 | 0.002 |
| $k_{LS}$ | mmol $l^{-1}$ | ß.1 | 0.035 |
| $Y_{X/Glc}$ | $10^7$ cells $l^{-1}$ mmol$^{-1}$ | 7.2 | 7.2 |
| $Y_{X/Gln}$ | $10^8$ cells $l^{-1}$ mmol$^{-1}$ | 1.7 | 1.7 |
| $q_{LS,max}$ | $10^{-11}$ mmol cell$^{-1}$ $h^{-1}$ | 1.4 | 1.4 |
| $Y_{Lac/Glc}$ | - | 1.5 | 1.1 |
| $Y_{Amm/Gln}$ | - | 0.6 | 1 |

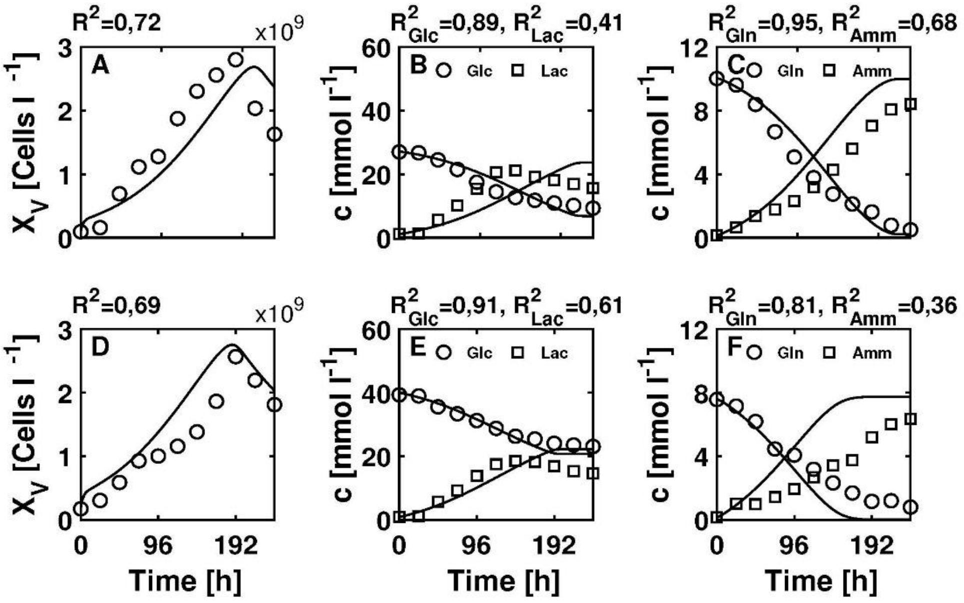


**Supplementary Figure 1** Growth curves of the cultivation in the shake flask (symbols) compared to the simulated growth curve (line). Plotted are cell numbers, substrates and metabolites against cultivation time. The MC concentration was 20 g $l^{-1}$. Data in graphs A, B and C are from experiment 1, Table 1, while D, E and F are from experiment 2, Table 1.

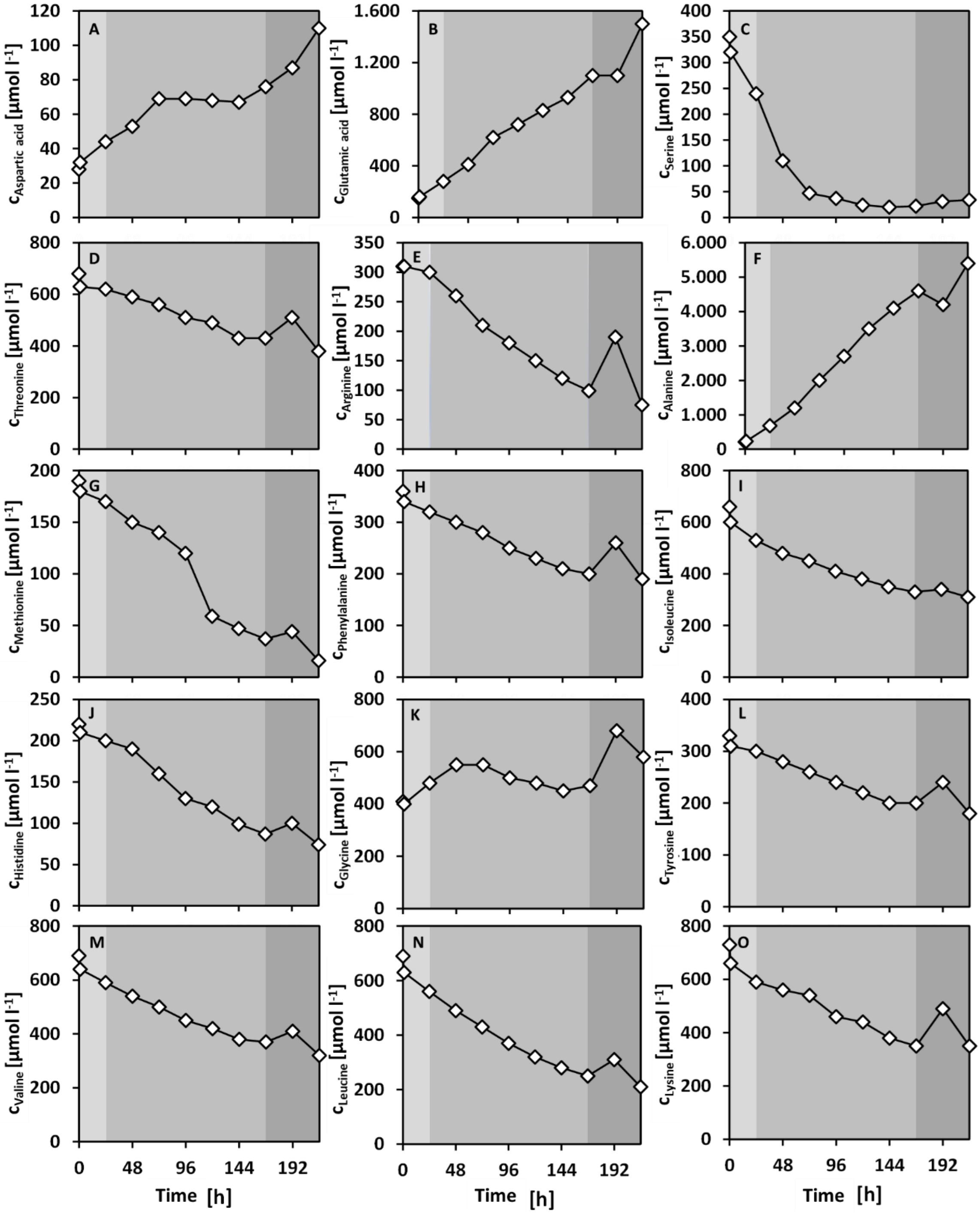


**Supplementary Figure 2** Diagrams of the 15 evaluated amino acids of experiment 1, Table 1. The colored background illustrates the attachment, the exponential phase and the death phase.

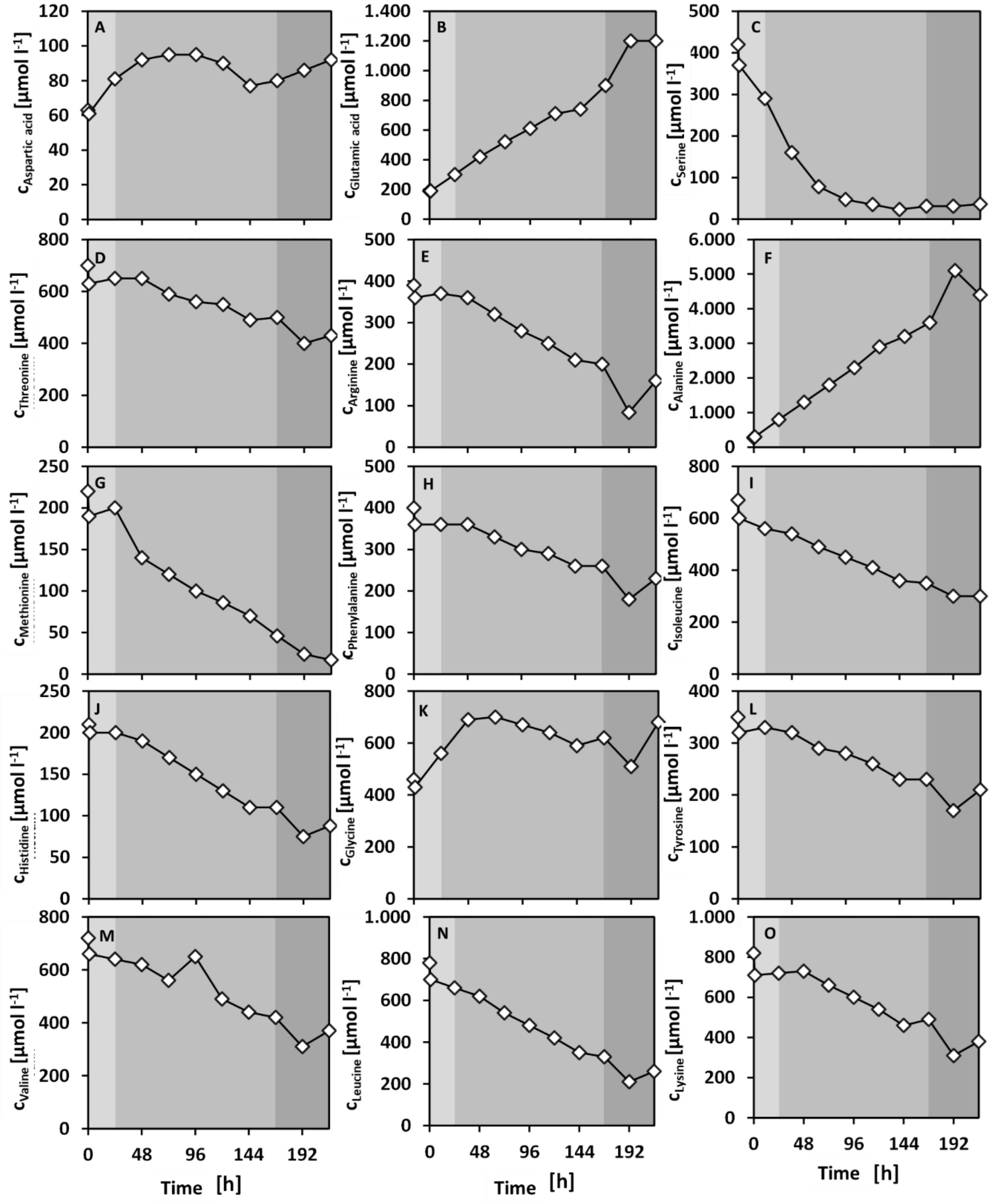


**Supplementary Figure 3** Diagrams of the 15 evaluated amino acids of experiment 2, Table 1. The colored background illustrates the attachment, the exponential phase and the death phase.

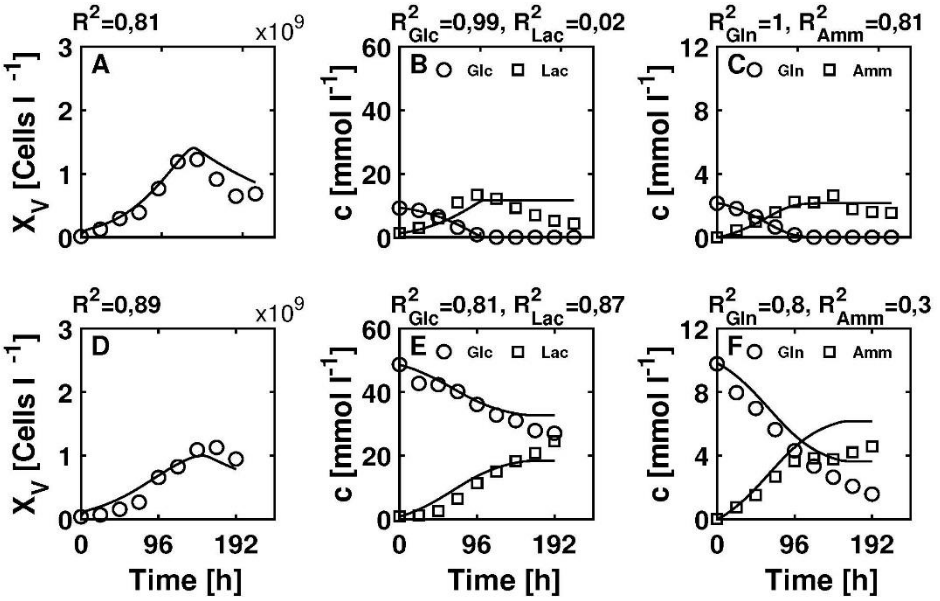


**Supplementary Figure 4** Growth curves of the cultivation in the shake flasks (symbols) compared to the simulated growth curve (line). Plotted are cell numbers, substrates and metabolites against cultivation time. The MC concentration was 10 g l$^{-1}$ (experiment 3, Table 1) and 5 g l$^{-1}$ (experiment 6, Table 1). Data in graphs A, B and C are from experiment 3, Table 1, while D, E and F are from experiment 6, Table 1.

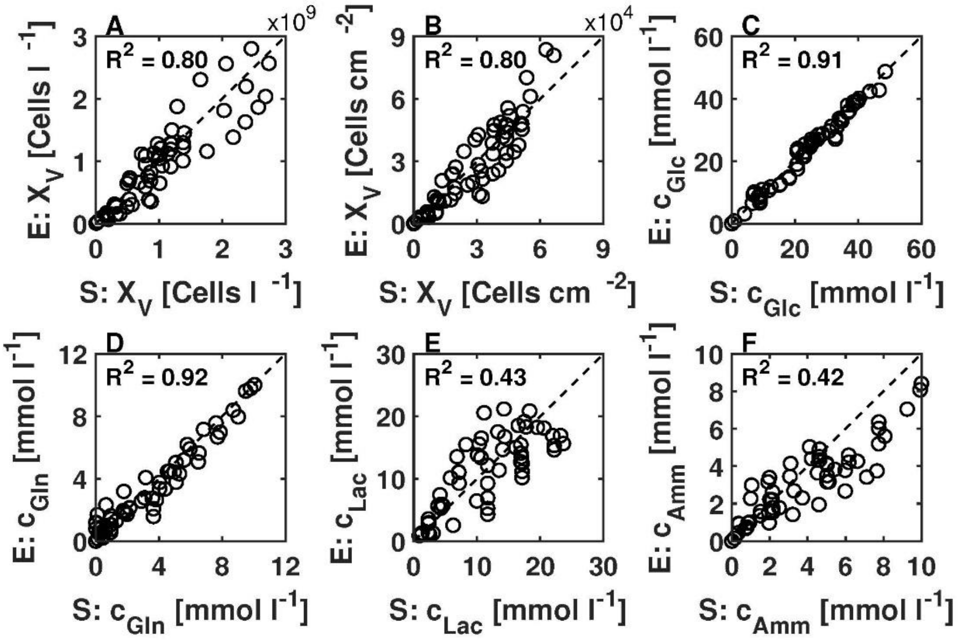


**Supplementary Figure 5** Growth curves of the cultivation in the shake flask in comparison to the simulated growth curve. The time course of the cell numbers and the time course of the substrate and metabolite concentrations (Glc, Gln, Lac, Amm) are shown.
